# Phytochemical Characterization and Antibacterial Activity of Albanian *Juniperus communis* and *Juniperus oxycedrus* Berries and Needle Leaves Extracts

**DOI:** 10.3390/antiox13030345

**Published:** 2024-03-13

**Authors:** Ilir Mërtiri, Bogdan Păcularu-Burada, Nicoleta Stănciuc

**Affiliations:** Faculty of Food Science and Engineering, Dunărea de Jos University of Galati, 111 Domnească Street, 800201 Galati, Romania; ilir_mertiri@yahoo.com (I.M.); bogdan.pacularu@ugal.ro (B.P.-B.)

**Keywords:** *Juniper communis*, *Juniper oxycedrus*, extracts, polyphenols, antibacterial activity

## Abstract

This paper aims to investigate the phytochemical profile and in vitro antibacterial activity of two juniper species collected in Albania, *Juniperus communis* and *Juniperus oxycedrus*. The berries and the needle leaves were subjected to solid–liquid solvent ultrasound-assisted extraction. The phytochemical characterization of the extracts was performed by spectrophotometric and chromatographic means. The extract of *J. communis* berries (JcB) showed a higher total phenolic and flavonoid content (3.04 ± 0.09 mg GAE/g DW, and 1.14 ± 0.36 mg QE/g DW, respectively), also a higher antioxidant activity from DPPH and ABTS radical screening assays, compared to *J. oxycedrus* berries (JoxB) extract. The extract of *J. oxycedrus* needle leaves (JoxL) prevailed in total flavonoid content (10.55 ± 0.24 mg QE/g DW), and ABTS assays (1.83 ± 0.01 mM TE/g DW), compared to the extract of *J. communis* needle leaves (JcL). The chromatographic analysis revealed the presence of ellagic acid and kaempferol in all the samples. Ellagic acid was the main identified compound with the highest quantity in the extracts of JoxB, JoxL, and JcB with an average of 445.69 ± 0.96 µg/g, 2890.05 ± 0.29 µg/g, and 8133.83 ± 4.03 µg/g, respectively. The antibacterial potential of the ethanolic extracts was evaluated on *Bacillus* spp., *Escherichia coli*, and *Staphylococcus aureus*. In the Agar Well Diffusion Assay, it was observed that all the tested bacterial strains were sensitive to the extracts, whereas selected extracts showed a similar inhibition activity rate compared with the antibiotic substance (Chloramphenicol), used as a positive control. The extracts showed a similar minimal inhibitory and bactericidal concentration for the individual bacterial strains, suggesting that *J. communis* and *J. oxycedrus* extracts have a similar potential in antibacterial activity.

## 1. Introduction

Plants contain a wide range of phytochemical compounds with diverse beneficial effects on human health, and the exploitation of this valuable source is oriented in several interrelated fields, such as the pharmaceutical, medical, cosmetic, and food industries [1,2]. The second metabolites have been associated with the antimicrobial activity of plants, while the low molecular weight compounds are classified into three main groups: alkaloids, terpenes, and phenolic compounds [3]. The European Medicines Agency has recognized *Juniper communis* L. berries and the related essential oils for corresponding antimicrobial, antiviral, antioxidant, and anti-inflammatory properties [4]. Juniper berries, essential oils, oleoresins (solvent-free), and natural extractives were also categorized as Generally Recognized as Safe (GRAS) for their intended use, in the Code of Federal Regulation in the US [5]. Juniper is a plant known since ancient times, mainly used in traditional medicine in the form of herbal teas, alone or combined with other plants for different remedies. In diverse cultures, juniper berries have found application in gastronomy, and also in alcoholic beverage production, such as gin or beer [6,7]. *Juniperus* are evergreen shrubs or trees and represent the most diverse genera of the conifers. The genus consists of approximately 75 species divided into 3 sections *Caryocedrus*, *Juniperus*, and *Sabina*. The section *Juniperus* is divided into 2 groups: a northern and far eastern group linked to *J. communis* and the other associated with *J. oxycedrus* spread in the Mediterranean region. *J. communis* is characterized by seed cones (known as “fruits” or “berries”), with a blue or blue-black color when mature, and one stomatal band on the adaxial leaf surface. This species is the most invasive in the *Juniperus* sect and the only one adopted in both hemispheres. The color of the mature berries of *J. oxycedrus* varies from red, red-coper, reddish-brown to reddish-purple, and the leaves have two stomatal bands [8]. In Albanian territory, a wide variety of juniper species such as *J. communis*, *J. oxycedrus*, *J. excelesa*, *J. foetidissima*, *J. macrocarpa*, *J. phoenicea,* and *J. sabina* can be found. *J. communis* is the most widespread of the species throughout the country, from the coastal areas to high altitudes. It grows mostly on Mount Korab, Lurë, the mountain range of Skanderbeg, Dajt, Librazhd, Polis-Vilë, Krrabë, Mokër, Gropë-Opar, Gramoz, the mountain range of Nëmërçkë-Trebeshinë-Dhembël, Kurvelsh, Çika mountain, Karaburun, and, rarely, in Delvinë and Saranda. *J. oxycedrus* grows at altitudes 1000–1100 m above sea level and is often found on the coast of Shëngjin, Kavaja, Fier, Vlora, and Saranda. More rarely, it is encountered in the interior part of the country such as Mat, Librazhd, Skrapar, Zagori, and Kurvelesh [9]. In 2020, *J. communis* and *J. oxycedrus* berries were on the list of the main Albanian Medical and Aromatic Plants (MAPs) for their increased collection due to the high demand from the exporting and essential oil production companies [10]. Juniper berries’ composition consists of essential oils, inverted sugars, resin, catechin, organic acid, terpenic acids, leucoanthocyanidin, flavonoids, tannins, gums, lignins, wax, etc. According to the study of different juniper extracts, concentrated in phenolic compounds, alkaloids, flavonoids, and tannins, they have been reported to possess high-to-moderate bacterial growth inhibition properties [7]. The decrease in efficiency and rising concern regarding the application of synthetic substances, mainly related to antibiotic-resistant bacteria, have awoken the interest among research groups to investigate and utilize MAPs as substitutes. A synthetic substance is usually composed of a single main bioactive compound, while MAPs’ essential oils or extracts provide a wider range of bioactive compounds with a synergism mechanism against the application target [11].

Conventional extraction methods (maceration, Soxhlet extraction, and hydro distillation), and more advanced extraction technologies (ultrasound, microwave, pulsed electric fields, enzymatic, and super- and sub-critical fluid) are used to separate bioactive compounds from plants. Conventional methods have standard extraction processes; however, they require a high quantity of solvent and a long extraction period, while advanced extraction methods show advantages related to the application of a lower amount of solvent, shorter extraction period, higher extraction efficacity, and the possibility to avoid toxic solvents, causing them to be considered as greener extraction methods [12,13].

The purpose of this study was to evaluate and compare the phytochemical composition, antioxidant, and antibacterial potential of two juniper species *J. communis* and *J. oxycedrus*, collected in wild conditions in Albania. A comparison is made between the berries and the needle leaves extracts, respectively. From the perspective of using the resulting extracts in food products to enhance the shelf life, a green extraction method and solvent were used to extract the bioactive compounds from the plant samples. The extracts were obtained using a solid–liquid solvent ultrasound-assisted extraction method; and, to assist the antibacterial activity, standard laboratory testing control strains were chosen, representing threats in the food industry.

## 2. Materials and Methods

### 2.1. Plant Materials

The wild juniper berries and needle leaves were collected in Albania between December 2022–January 2023. *J. communis* samples were collected in Mat; Dibër (41°28′11.3″ N 20°02′24.2″ E), while *J. oxycedrus* samples were collected in Has; Kukës (42°16′44.0″ N 20°22′15.1″ E). Samples were identified with the help of Prof. Dr. Lulëzim Shuka (Department of Biology; Faculty of Natural Science; University of Tirana; Albania). The drying process of the samples was carried out in room conditions. The moisture content and water activity were measured using a moisture analyzer (KERN; DAB 100-3; Balingen, Germany) and a water activity meter (Fast-lab; GBX Scientific Ltd.; Romans sur Isére Cédex, France).

### 2.2. Reagents

Reagents for the determination of total phenolic content (TPC), total flavonoid content (TFC), total anthocyanin content (TAC), antioxidant radical screening reagents [2,2-Diphenyl-1-picrylhydrazyl (DPPH), and 2,2′-azino-bis(3-ethylbenzothiazoline-6-sulfonic acid) (ABTS)], standards for HPLC identification and quantification, and microbiological media/broth (Brain Heart Infusion Borth, Nutrient Agar Media), were purchased from Sigma-Aldrich (Steinhelm, Germany). Ethanol, and acetonitrile of HPLC grade were purchased from Honeywell (Seelze, Germany). Methanol and glacial acetic acid of analytical grade were secured from S.C. Chimreactiv, S.R.L. (Bucharest, Romania), while the Folin–Ciocâlteu reagent from Remed Prodimpex S.R.L. (Bucharest, Romania). Chloramphenicol was purchased from Thermo Scientific™ Oxoid™ (Hampshire, UK).

### 2.3. Ultrasound-Assisted Extraction

The dried juniper berries and needle leaves were ground in an electric grinder (Heinner HCG-150SS; Bucharest, Romania) and extracted using a Digital Ultrasonic Bath (Mod. DU-32; ARGOLAB; Capri, Italy). Ethanol is one of the most used solvents to extract bioactive compounds from plant material. Therefore, the leaves were extracted using ethanol 70% (*v*/*v*) [14], while, for the berries, an organic acid like acetic acid (considered safe for consumption) was added to modify the polarity of ethanol, expanding the range of the extracted compounds and protecting sensitive photochemical compounds from oxidation [15]. The extraction solution applied to berries consisted of a mixture of ethanol 70% (*v*/*v*) and acetic acid (9:1 *v*/*v*). The conditions used for the ultrasound-assisted extraction were a triple-stage, batch extraction at 25 °C, 15 min, 40 kHz, and 1:10 (*w*/*v*) plant:solvent ratio. These parameters were chosen to extract thermo-sensitive bioactive compounds. After the extraction process, the collected extracts were centrifuged at 6500 rpm, 15 min, 4 °C, and the supernatant was concentrated using a Rotary Vacuum Concentrator (RVC 2-18 CDplus, Martin Christ; Osterode am Harz, Germany) equipped with a vacuum pump and cooling trap (CT02-50, Martin Christ; Osterode am Harz, Germany). After the concentration process, the samples were stored at 4 °C until further analyses. The extraction yield was expressed in percentage and was calculated based on the weight of the sample used for extraction and the weight of the extract obtained after the concentration process.

### 2.4. Determination of Total Phenolic Content (TPC), Total Flavonoid Content (TFC), and Total Anthocyanin Content (TAC) of the Extracts

The concentrated extracts were redissolved in ethanol 70% (*v*/*v*), the phytochemical characterization and antioxidant activity were estimated by colorimetric means (Biochrom; Libra 22 UV/Visible Spectrophotometer; Holliston, MA, USA), in triplicate tests. The protocol used for the measurement of the total phenolic content (TPC) involved the Folin–Ciocâlteu reagent and Na_2_CO_3_ 20% (*w*/*v*), as described in detail by Serea et al. [16]. The reading of absorbance was performed at 765 nm. The results are expressed in milligrams of gallic acid equivalents per gram of dry weight (mg GAE/g DW).

The determination of total flavonoid content (TFC) was based on the description of Yangui et al. [17], with some modifications. Briefly, a volume of 0.25 mL AlCl_3_ 2% (methanolic solution *w*/*v*) was added to 0.25 mL of extract, followed by the addition of 1.5 mL methanol. The mixtures were homogenized and kept in the dark for 15 min, and the reading of absorbance was performed at 440 nm. The results are expressed in milligrams of quercetin equivalents per gram of dry weight (mg QE/g DW).

The total anthocyanin content was determined according to the description of Sethi et al. [18]. Thus, a volume of 0.2 mL of extract was combined with 0.8 mL KCl (0.025 mol, pH = 1.0) and simultaneously another 0.2 mL of extract with 0.8 mL CH_3_COONa (0.4 mol, pH = 4.5). The mixtures were homogenized and kept in darkness for 15 min; the absorbance was read at 520 nm and 700 nm. The results are calculated using Equation (1) and expressed in micrograms of cyanidin 3-glucoside equivalents per gram of dry weight (µg C3GE/g DW).

(1) $$\mathrm{TAC}\;\left(µg\;C3\mathrm{GE}/g\;\mathrm{DW}\right)=\frac{\left[\mathrm{pH}1.0\;\left(A_{520}-{\;A}_{700})-\;\mathrm{pH}4.5\;(A_{520}-{\;A}_{700}\right)\right]\times \;\mathrm{MW}\;\times \;V\;\times \;\mathrm{DF}\;}{\varepsilon \;\times \;m}$$

where: A—the measured absorbance; MW—molecular weight of cyanidin 3-glucoside (449.38 g/mol); V—the volume of the analyzed extract (µL); ε—molar absorptivity of cyanidin 3-glucoside (26,900 L mol^−1^ cm^−1^); m—the weight of the concentrated extract (g); DF—dilution factor.

### 2.5. Antioxidant Activity of the Extracts

The DPPH radical scavenging assay was based on the protocol described by Labri et al. [19], with some minor modifications. A volume of 0.1 mL of the sample was added to 3.9 mL of DPPH (4 mg of DPPH dissolved in 100 mL of methanol), homogenized, and left in darkness for 30 min. The reading of absorbance was performed at 515 nm. The protocol that followed for the ABTS radical scavenging assay was described by Dumitrașcu et al. [20]. Briefly, a volume of 0.02 mL extract was added to 1.980 mL of ABTS (7 mmol), the mixture was homogenized and left in the dark for 20 min. The reading of absorbance was performed at 734 nm. The results are expressed in mmol of Trolox equivalents per gram of dry weight (mmol TE/g DW).

### 2.6. Chromatographic Analysis of the Extracts

The phenolic profile of the extracts was analyzed using an Agilent 1200 HPLC system equipped with an autosampler, degasser, quaternary pump system, multi-wavelength detector, and column thermostat (Agilent Technologies; Santa Clara, CA, USA). The separation conditions are described in detail in previously published works [21]. Briefly, for the separation of the phenolic compounds in the ethanolic extracts, a binary elution system of 1% acetic acid (*v*/*v*) as solvent A and 100% acetonitrile (*v*/*v*) as solvent B was used, followed by reading the absorbance at 280 and 320 nm. Results are expressed as average values for duplicate measurements ± standard deviation, in micrograms per gram extract (µg/g extract).

### 2.7. In Vitro Antibacterial Activity

#### 2.7.1. Microorganisms Used

The strains used to perform the antibacterial activity investigation were *Escherichia coli* (ATCC 25922), *Staphylococcus aureus* (ATCC 25923), and *Bacillus* spp. (spore-forming bacterial strain) from the Microorganisms Collection of “Dunărea de Jos” University of Galați (MIUG). The bacterial strains were reactivated in sterile Brain Heart Infusion (BHI) Broth and incubated overnight at 37 °C. The number of colony forming units (CFU) of the overnight culture was measured by optical density at 600 nm (JENWAY Spectrophotometer; Model 6505 UV-Vis; Great Dunmow, UK) until the working dilution.

#### 2.7.2. Agar Well Diffusion Assay

The concentrated extracts were dissolved in a mixture of 25% acetone (*v*/*v*), and 1% Tween-80 (*v*/*v*). This mixture composition was chosen to improve the solubility of the extracts, as suggested by Masota et al. [22]. The tested concentration chosen to perform the antibacterial assessment for the juniper extracts was 50 mg/mL, the lowest concentration used in the work of Hannan et al. [23]. The solubilized extracts were filtered in sterile conditions using Nylon Syringe Filters (0.22 µm). The Agar Well Diffusion Assay was based on the method described by Farahmandfar et al. [24], with some modifications. The assay was performed using the Pour Plate Method; a volume of 1 mL of 2 × 10^8^ CFU/mL inoculum was mixed with 15–20 mL of Nutrient Agar Media at 45 °C (a final concentration of 10^7^ CFU/mL). After solidification, the wells in the media were created using a sterile glass test tube (11 mm diameter), and 0.1 mL of extract was added to the well. Chloramphenicol (1 mg/mL) was used as a positive control and the solubilization reagent (25% acetone, and 1% Tween-80) as a negative control. The Petri dishes remained for 20 min in the laminar flow chamber for the samples to diffuse in the media before being placed in the incubator at 37 °C for 24 h. The results for duplicate tests are expressed as mean ± SD, in millimeters for Diameter Inhibition Zone (mm DIZ).

#### 2.7.3. Minimum Inhibitory Concentration and Minimum Bactericidal Concentration

The Minimum Inhibitory Concentration (MIC) protocol was based on the description of Alshareef [25] and Parvekar et al. [26]. In a 96-well microtiter plate, 100 µL of BHI Broth was placed on each well using a multichannel pipette. A total of 100 µL of the extract was placed in the first well of the row. A serial dilution was performed by drawing 100 µL of the mixture of BHI Broth and sample extract from the first well and placing it in the second. The process was repeated until the tenth well; on the last well, the excess volume was disposed to have the same volume in all the tested wells. A total of 10 µL of 10^6^ CFU/mL of inoculum was added to each well, followed by 10 µL of sterile filtered Resazurin (2 mg/mL). Chloramphenicol (1 mg/mL), dissolved in ultrapure water, was used as a positive control, and the solubilization reagent (25% acetone and 1% Tween-80) as a negative control. The microplates were incubated at 37 °C for 24 h. The investigation of Minimum Bactericidal Concentration (MBC) was performed based on the method described by Parvekar et al. [26], with some minor modifications. The 10 µL from the MIC wells that did not show visible bacterial growth were inoculated in Petri dishes with solidified Nutrient Agar Media and incubated at 37 °C; the results were observed after 12–24 h.

### 2.8. Statistical Analysis

The results are expressed as average values for duplicate or triplicate measurements ± standard deviation (SD). The statistical evaluation was performed in Minitab Software for Windows, Version 19.1. The differences between the extracts of the berries and the leaves of the two juniper species were analyzed using the ANOVA method. The data were checked for the normality distribution (Ryan-Joiner Test) and the equality of the variances (Bartlett’s Test), followed by the Tukey test (*p* > 0.05) or the Games–Howell test (*p* < 0.05), and 95% confidence.

## 3. Results

### 3.1. Extraction Yield of the Extracts

The results of the extraction yield obtained from the juniper samples are shown in Table 1. From the ultrasound extraction with 70% ethanol and acetic acid, a similar extraction yield was observed for both JcB and JoxB berries, respectively, at 54.21% and 54.40%. However, in the 70% ethanolic extraction from the needle leaves, JoxL showed a higher extraction yield of 30.19%, compared with 24.20% from JcL.

### 3.2. Phytochemical Characterization and Antioxidant Activity of the Extracts

Table 2 presents the results from the phytochemical characterization of the juniper berries and needle leaves using spectrophotometric means. Based on statistical evaluation, JcB showed a significantly higher phenolic and flavonoid content (*p* > 0.05), respectively, at 3.04 ± 0.09 mg GAE/g DW and 1.14 ± 0.36 mg QE/g DW, compared with JoxB. For the needle leaves, both samples did not show any significant differences in the total phenolic content (*p* > 0.05); however, JoxL showed higher flavonoid content with an average of 10.55 ± 0.24 mg QE/g DW. JoxB showed a higher content of anthocyanin with 79.03 ± 0.00 µg C3GE/g DW. In both antioxidant assays, JcB presented a higher activity with an average of 0.07 ± 0.01 mM TE/g DW for the DPPH radical screening assay and 0.21 ± 0.01 mM TE/g DW for the ABTS radical screening assay. For both juniper needle leaves extracts, no significant differences in the antioxidant activity of the DPPH assay were observed; however, in the ABTS assay, JcL showed a higher antioxidant activity compared to the JoxL extract, respectively, at 1.83 ± 0.01 mM TE/g DW and 1.67 ± 0.01 mM TE/g DW. The antioxidant reaction mechanisms in both assays, DPPH and ABTS, consist of single electron transfer. The reaction rate in each assay depends on different physical processes, where the dominant reaction depends on the pH and solvent, resulting in different structure–activity relationships for each assay. The antioxidant activity also depends on the reaction with the functional group of the different classes of phenolic compounds [27].

### 3.3. HPLC Phytochemical Profile of the Extracts

**Figure 1:**
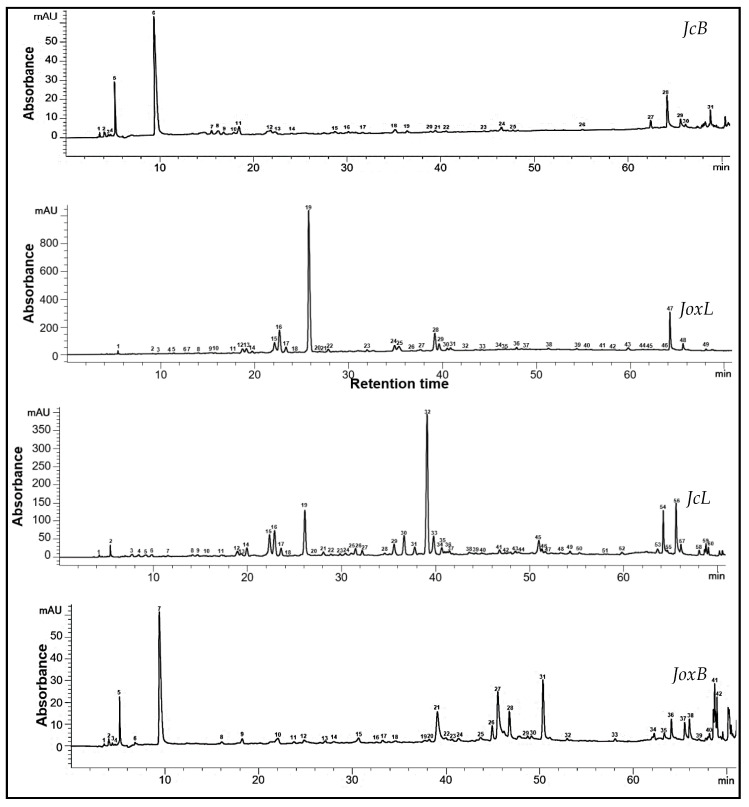
HPLC chromatogram of the samples at 280 nm. Identified peaks in JcB: 5—theaflavin, 7—protocatechuic acid, 12—catechin, 15—syringic acid, 20—ellagic acid, 21—epicatechin gallate, 22—quercetin 3-D-galactoside; JoxB: 4—theaflavin, 7—gallic acid, 11—chlorogenic acid, 13—vanillic acid, 18—procyanidin A1, 21—ellagic acid, 24—quercetin 3-glucoside, 33—luteolin, 35—kaempferol; JcL: 5—gallic acid, 10—protocatechuic acid, 18—caffeine, 20—vanillic acid, 21—caffeic acid, 22—syringic acid, 28—procyanidin A1, 32—ellagic acid, 35—ferulic acid, 36—quercetin 3-glucoside, 39—naringin, 40—hesperidin, 51—luteolin, 53—kaempferol; JoxL: 9—protocatechuic acid, 11—(−)—epigallocatechin, 18—caffeine, 20—vanillic acid, 21—caffeic acid, 22—syringic acid, 24—procyanidin A1, 26—p-coumaric acid, 28—ellagic acid, 30—quercetin 3-D-galactoside, 31—ferulic acid, 32—quercetin 3-glucoside, 33—naringin, 42—luteolin, 46—kaempferol.

**Figure 2:**
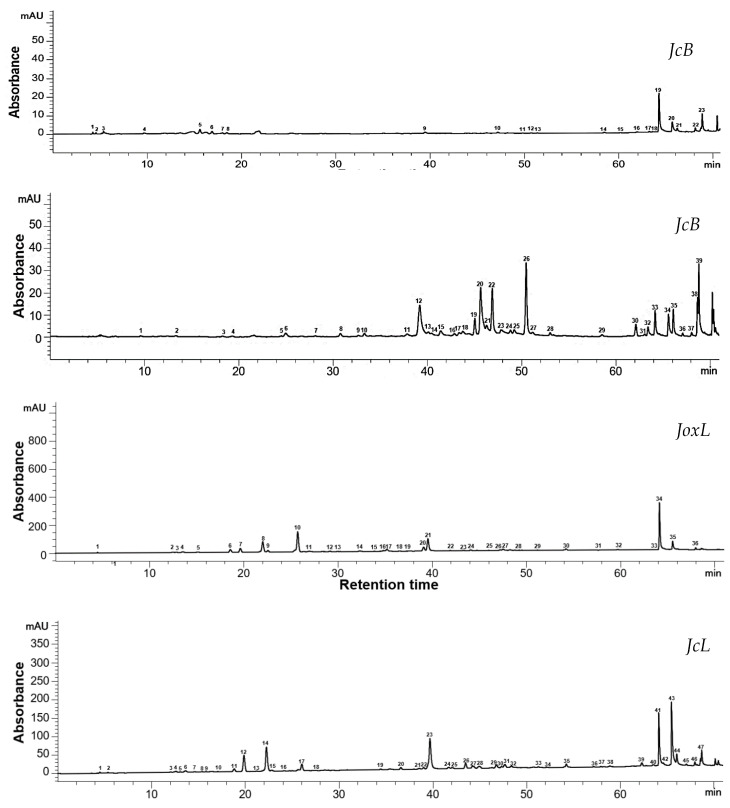
HPLC chromatogram of the samples at 320 nm. Identified peaks in JcB: 9—epicatechin gallate, 17—apigenin, 18—kaempferol; JoxB: 1—gallic acid, 14—quercetin 3-D-galactoside, 15—quercetin 3-glucoside, 25—myricetin, 31—apigenin, 32—kaempferol; JcL: 18—caffeic acid, 24—quercetin 3-β-D-glucoside, 37—luteolin, 42—kaempferol; JoxL: 19—p-coumaric acid, 22—quercetin 3-glucoside, 28—myricetin, 31—luteolin, 33—kaempferol.

The results of the HPLC identification and quantification are shown in Table 3, whereas the typical chromatograms are given in Figure 1 and Figure 2. For JcB, catechin showed the highest content with an average of 93.98 ± 0.37 µg/g extract, without quantifiable levels in the other samples. The main polyphenolic compound in JoxB was ellagic acid with an average concentration of 445.69 ± 0.96 µg/g of extract, followed by gallic acid (290.23 ± 0.13 µg/g extract). Ellagic acid predominates in both needle leaves extracts, accounting for 8133.83 ± 4.03 µg/g extract in JcL, and 2890.05 ± 0.29 µg/g extract in JoxL, followed by kaempferol (179.87 ± 2.26 µg/g extract) and (−)—epigallocatechin (1129.23 ± 3.66 µg/g extract). The common bioactives identified in both berries and needle leaves extracts were kaempferol and ellagic acid, with a higher level in the needle leaves extracts. Apigenin and theaflavin were detected only in the berry extracts, with a higher content in JoxB of 3.17 ± 0.01 µg/g extract and 3.75 ± 0.02 µg/g extract, respectively. The presence of caffeic acid, caffeine, and naringin was detected only in the needle leaves extracts. Caffeic acid and caffeine showed a higher content in JoxL (85.98 ± 0.28 µg/g extract, and 12.44 ± 0.08 µg/g extract, respectively), while naringin a higher quantity in JcL with an average of 12.13 ± 0.06 µg/g extract. Hesperidin was detected only in the JcL (6.04 ± 0.95 µg/g extract), and myricetin in the *J. oxycedrus* samples, berries and needle leaves (28.24 ± 4.57 µg/g extract, and 22.15 ± 0.19 µg/g extract, respectively).

As can be observed in the chromatograms in Figure 1 and Figure 2, the JoxB extract showed higher responses caught by the detector, with 42 recorded peaks at 280 nm and 39 peaks at 320 nm. The higher number of peaks recorded suggests a richer profile of the extract with phenolic compounds, also conformed from the higher number of identified compounds in the extract of JoxB compared to JcB, with 12 and 9 identified peaks, respectively. In the chromatograms of needle leaves extracts, JcL showed a richer phenolic profile with 60 recorded peaks at 280 nm and 47 peaks at 320 nm caught by the detector. However, several peaks remained unidentified due to the absence of satisfying correspondence in the standards HPLC system database at our research center.

### 3.4. Antibacterial Activity

In general terms, Chloramphenicol, used in this work as a positive control, has a wide spectrum of antibiotic activity against Gram-positive, Gram-negative, and anaerobic bacteria harmful to humans and animals. Its antibiotic mechanism consists of protein-forming inhibition, causing the death of the bacteria. In some countries, like the USA, Canada, European Union, China, Japan, and Australia, its application is limited or banded due to some side effects like aplastic anemia (disease that causes red and white blood cell production deficiencies), and potentially genotoxic carcinogenicity [28,29]. Therefore, novel natural molecules and extracts are needed to replace the use of synthetic compounds.

#### 3.4.1. Agar Well Diffusion Assay

**Figure 3:**
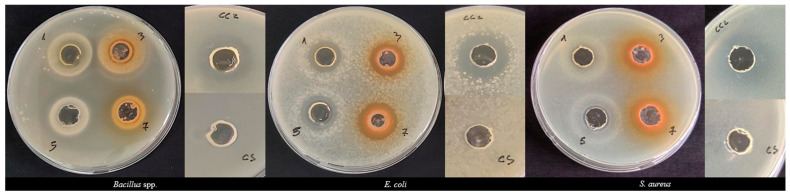
Petri dishes with the results from the Agar Well Diffusion Assay for the tested bacterial strains. The number–sample coded on the wells represents: 1—JcB; 3—JcL; 5—JoxB; 7—JoxL; CC2—Chloramphenicol (positive control); CS—solubilization solvent (negative control).

Table 4 and Figure 3 show the results obtained from the Agar Well Diffusion Assay against the tested bacterial strains. Both berries and needle leaves extracts (50 mg/mL extract), compared with the positive control Chloramphenicol (1 mg/mL), did not show significant statistical differences in the inhibition activity against *Bacillus* spp. For *E. coli*, only JcB showed a lower diameter of inhibition zone, with an average of 17.50 ± 0.71 mm, compared with the positive control (DIZ: 22.00 ± 0.71 mm). Regarding the statistical evaluation, both berries’ extracts showed a similar inhibition activity compared to Chloramphenicol against *S. aureus* (respectively 26.00 ± 1.41 mm for JcB, and 26.50 ± 2.12 mm for JoxB), while the needle leaves extracts showed a lower inhibition zone than the control against *S. aureus* (18.50 ± 0.71 mm for JcB extract and 19.50 ± 0.71 mm for JoxB extract).

Comparing the inhibition activity of the individual extracts against the different tested strains, JoxB extract showed a higher inhibition effect on *S. aureus* compared to the other two bacterial strains. Meanwhile, the JoxL extract had a higher inhibition effect on *E. coli* (DIZ: 20.75 ± 0.35 mm), and a lower inhibition activity on *Bacillus* spp. (DIZ: 17.50 ± 0.71 mm). From the statistical evaluation of the *J. communis* berries extract, among the different tested strains, no significant differences in the inhibition rate were observed. The same phenomena were noticed for the *J. communis* needle leaves extract.

#### 3.4.2. Minimum Inhibitory Concentration and Minimum Bactericidal Concentration

From Table 5, it can be observed that both juniper species’ berry extracts have a similar minimal inhibitory and bactericidal concentration. The extracts showed a MIC of 0.05 mg/mL for *Bacillus* spp. and 6.25 mg/mL for *E. coli* and *S. aureus*. The MBC for all the tested strains was 12.50 mg/mL. *J. communis* and *J. oxycedrus* needle leaves extracts showed a MIC of 25.00 mg/mL against *S. aureus* but it was not possible to detect the MIC and MBC for *Bacillus* spp., *E. coli*, and the MBC for *S. aureus*. However, based on the results obtained from the Agar Well Diffusion Assay, the minimal concentration for the inhibitory and bactericidal effect can be estimated to be higher than 25.00 mg/mL, which represents the highest tested concentration performed in the assessment of MIC and MBC. The results obtained from the MIC and MBC are correlated to the data obtained from the Agar Well Diffusion Assay. The berries extracts showed a similar antibacterial activity both for berries and needle leaves extracts.

## 4. Discussion

Belov et al. [14] reported an extraction yield of 8.79 ± 0.15% from the ethanolic ultrasound-assisted maceration of *J. communis* berries. Additionally, Meringolo et al. [30] used the methanolic extraction of *J. macrocarpa* and *J. oxycedrus* needle leaves and reported an extraction yield of 10.4% and 10.8%, respectively. Orhan et al. [31], reported the ethanolic extraction from different juniper species (*J. oxycedrus* subs. *oxycedrus*, *J. communis* subsp. *nana*, *J. sabina*, *J. foetidissima*, and *J. excelesa*), an extraction yield varying between 27.6–35.2% for the needle leaves, and between 19.8–36.2% for the ripe berries. The results reported by Orhan et al. [31] were in a close yield range. However, considering the extraction time used, including a double-stage maceration of an 8 h period, the ultrasound-assisted extraction performed in this study allows for a shorter extraction time and provides economic efficiency from the perspectives of industrial scale adaptation. The extraction yield obtained from the juniper berries in our study was higher compared to the results available in the literature for the same botanical plant. These differences in the extraction yield may be attributed to extraction conditions such as plant–solvent ratios, ultrasound frequency, and the use of acetic acid to increase the solvent polarity and extracted phytochemical range.

The higher extraction yield may have contributed to a lower expression of total phenolic and flavonoid content in the spectrophotometric characterization. Different authors and works have reported higher phenolic and flavonoid content in the needle leaves and berries of different juniper species. Popescu et al. [32], reported a total phenolic content that varied between 12.67 ± 0.01 and 24.77 ± 0.02 for the ethanolic maceration of mature *J. communis* berries collected in three different locations in Romania during a 3-month period. The ethanolic extracts of *J. communis* berries collected in Slovakia, for 3 years showed a total phenolic content varying between 6.87 ± 0.47–42.23 ± 2.62 GAE mg/g DM (dry matter) [33]. Belov et al. [14] reported for *J. communis* berries, from the first and the second year of maturation, total phenolic content of 44.31 ± 0.33 mg GAE/g DE (dry extract), and 10.84 ± 0.15 mg GAE/g DE, respectively, and total flavonoid content of 40.33 ± 0.33 mg QE/g DE and 9.37 ± 0.37 mg QE/g dry extract, respectively. Methanolic extracts of *J. oxycedrus* needle leaves and berries collected in Morocco have shown a TPC of 292.52 ± 11.68 mg GAE/g DW and 131.48 ± 4.58 mg GAE/g DW, respectively, and TFC of 54.58 ± 2.98 mg QE/g DW and 8.28 ± 0.74 mg QE/g DW [34]. There have been reports in the literature showing lower total phenolic content of 0.19 ± 0.01 mg GAE/g extract reported for hydroalcoholic extract of *J. communis* berries collected in Romania [35]. Phenolic compounds are secondary metabolites and have diverse functions in plants such as protection against herbivores, pathogen, oxidative stress, UV light, a form of communication with other plants, and structural roles. Their production is related to the plant needs, depending on the biotic and abiotic stress [36]. The high differences in the total phenolic and flavonoid content observed from our spectrophotometric characterization and the data in the literature may be attributed to environmental conditions, the harvest period, extraction conditions, and the protocol assessment.

In terms of antioxidant potential, the obtained extracts showed higher values for antioxidant activity when compared with methanolic extracts from *J. sabina* needle leaves (0.09–1.56 mmol Trolox/g DW on ABTS basis and 8.7 ± 1.2–2129.5 ± 88.4 µmol Trolox/100 g DW on DPPH basis) [37]. However, these authors reported a higher concentration in TPC (388.39 ± 24.49–611.27 ± 91.04 mg GAE/g DW). The differences in the antioxidant activity values may be attributed to the selectivity of the solvent, the extraction conditions, and the antioxidant potential of the extracted compounds.

The HPLC analyses of the ethanolic extracts of *J. communis* berries, collected in three different locations in Romania during 3 months [32], showed a higher content of ellagic acid (0.6 ± 0.1–2.3 ± 0.3 µg/g DW), catechins (7298.7 ± 0.2–9898.9 ± 0.5 µg/g DW), quercetin (924.5 ± 0.1–1100.5 ± 0.2 µg/g DW), and kaempferol (4001.2 ± 0.3–5991.3 ± 0.1 µg/g DW), when compared with the content found in the JcB extract (0.02 ± 0.00 µg/g extract for ellagic acid, 93.98 ± 0.37 µg/g extract for catechins, 10.83 ± 0.40 µg/g extract for kaempferol, and 6.07 ± 0.08 µg/g extract for quercetin 3-D-galactoside). In this study, the JcB extract, chlorogenic acid, gallic acid, vanillic acid, epicatechin gallate, ferulic acid, and caffeic acid were not detected. However, the Romanian juniper berry extracts contained 10.2 ± 0.2–11.1 ± 0.2 µg/g DW chlorogenic acid, 0.1 ± 0.0 µg/g DW gallic acid, 0.2 ± 0.0 µg/g DW vanillic acid and epicatechin gallate, 72.3 ± 0.5–94.5 ± 0.4 µg/g DW for caffeic acid, and 0.1 ± 0.1–0.8 ± 0.1 µg/g DW ferulic acid. The Albanian JcB extract showed a higher content of syringic acid (5.75 ± 0.02 µg/g extract), compared to 0.9 ± 0.2–2.1 ± 0.2 µg/g DW found in the Popescu et al. [32].

From the reported HPLC-DAD results in the work of Meringolo et al. [30], in the methanolic extract of *J. oxycedrus* needle leaves, it was found that luteolin (329.6 ± 8.7 µg/g extract), syringic acid (26.71 ± 0.4 µg/g extract), and vanillic acid (65.3 ± 0.8 µg/g extract) showed a higher content when compared with JoxL. These compounds showed a lower content of 5.35 ± 0.66 µg/g extract, 8.62 ± 0.02 µg/g extract, and 23.21 ± 0.16 µg/g extract for luteolin, syringic acid, and vanillic acid, respectively. The presence of apigenin and chlorogenic acid was not detected in our JoxL extract; however, in the Italian *J. oxycedrus* needle leaves, these compounds were detected at a content of 324.8 ± 8.2 µg/g extract, and 246.2 ± 9.2 µg/g extract, respectively. In both works, kaempferol was detected at a similar quantity, with an average of 57.76 ± 9.47 µg/g extract in JoxL extract, and a total content of 57.2 ± 4.3 µg/g extract consisting of kaempferol and, kaempferol-3-O-glucoside. Gallic acid was not detected in both needle leaves extracts, while protocatechuic acid was detected at a content of 15.80 ± 0.75 µg/g extract in our JoxL extract.

In the HPLC-DAD characterization of an aqueous, and a methanolic extracts of Moroccan *J. oxycedrus* subsp. *oxycedrus* needle leaves, a content of 86.8–50 µg/g extract for caffeic acid, 175–212 µg/g extract for *p*-coumaric acid, 645–184 µg/g extract for naringenin, and 2788–2572 µg/g extract for hesperidin were reported [34]. Meanwhile, in the JoxL ethanolic extract, the same compounds were detected at a concentration of 85.98 ± 0.28 µg/g extract for caffeic acid, 12.73 ± 0.09 µg/g extract for *p*-coumaric acid, 8.26 ± 0.14 µg/g extract for naringenin, while the presence of hesperidin was not detected. Syringic acid (8.62 ± 0.02) was detected in JoxL ethanolic extract, while in the Mrid et al. [34] study, this compound was not detected in nether aqueous or the methanolic needle leaves extract.

As it has been reported, bioactive compounds identified in our study possess antioxidant activity (except caffeine), and selected bioactives showed anti-inflammatory properties. Several reports showed that caffeic acid, gallic acid, kaempferol, *p*-coumaric acid, protocatechuic acid, syringic acid, theaflavin, and vanillic acid have antimicrobial activity [38,39,40,41,42,43,44]. As it can be seen from the HPLC analysis, a wide profile related to the phytochemical composition was found, evidencing the differences and similarities in the *J. communis* and *J. oxycedrys* berries and needle leaves collected in different geographical locations. From the data obtained, the presence of theaflavin identified in both juniper berries, and myricetin in *J. oxycedrus* berries, has not previously been reported in the literature. Given the abovementioned results, it can be concluded that the bioactive profile of *Juniper* ssp. highly depends on the geographical region and the extraction method.

It is well known that medical plants, such as *Juniper* ssp., play an important role in food, pharmacology and medicine, especially due to their effectiveness, fewer side effects and a relatively low cost. Therefore, different studies reported the substantial effect of phenolic extracts on anticarcinogenic and antimutagenic activities [45]. For example, for the ellagic and curcumin, Hayeshi et al. [45], reported a protective effect of these two compounds on the inhibition of over-expressed glutathione S-transferases, which are multifunctional detoxification proteins that protect the cell from electrophilic compounds.

However, the further application of both extracts’ cytocompatibility depends on the application field. As explained above, the higher content of ellagic acid may recommend these extracts in medicine and pharmacological fields. However, in our study, we were focused on food field applications as antimicrobials to extend the shelf life of the foods.

According to Ertürk [46], the MIC for *B. subtilis* (ATCC 6633), *E. coli* (ATCC 25922), and *S. aureus* (ATCC 25923) were 5 mg/mL for the *J. oxycedrus* berries extracted by maceration in ethanol 70%. Esteban et al. [47] reported a MIC of 2.5 mg/mL for *E. coli* and *S. aureus* (MRSA) methicillin-resistant from the essential oils obtained from *J. communis* foliage biomass containing branches, twigs, leaves, and berries collected in Lubia (Spain). The difference between the minimal inhibition and bactericidal concentration obtained in this study may be attributed to important factors like the harvesting season and location influencing the antibacterial activity of plant extracts [48]. Semerdjieva et al. [49,50], reported that *S. aureus* subs. *aureus* (CCM 4223) was the most sensitive bacterial strain when using essential oils extracted from berries and branches with needle leaves of the *J. oxycedrus*. Being a Gram-positive bacteria, *S. aureus* is more sensitive to antibiotic compounds due to the cell wall structure [51]. Our results are in alignment with the reported data, highlighting the antibacterial activity of the extracts or the essential oils obtained from the needle leaves and/or berries of *J. communis* and *J. oxycedrus* against *E. coli*, *S. aureus* and *Bacillus* strains (most often *Bacillus cereus* and *Bacillus subtilis*) and other bacterial strains [46,47,49,50,52,53].

## 5. Conclusions

In this study, a phytochemical characterization of *J. communis* and *J. oxycedrus* berries and needle leaves collected in Albania was performed through spectrophotometric means, HPLC analyses, and in vitro antibacterial activity. To employ a comprehensive profile, in terms of phenolic profile, the samples were extracted by solid–liquid ultrasound-assisted method. The addition of an organic acid like acetic acid to ethanol 70% for the extraction of the berries’ bioactive compounds led to a higher extraction yield. The ethanolic extract of *J. communis* berries showed a higher total phenolic and flavonoid content, consequently contributing to a higher antioxidant activity compared with the *J. oxycedrus* berries extract, which prevailed in the total anthocyanin content. The needle leaves extracted from *J. oxycedrus* showed better results in total flavonoid content and antioxidant activity. Ellagic acid and kaempferol were the phytochemical compounds, identified in all the extracts. Ellagic acid showed the highest amount in *J. oxycedrus* berries and needle leaves, whereas the main bioactive compound found in *J. communis* berries was catechin. Both juniper berry extracts contained apigenin and theaflavin, and, in *J. oxycedrus* berries’ myricetin, compounds previously not reported in the literature for these juniper species. In the antibacterial activity assays, it was observed that all of the juniper extracts showed inhibition on the tested bacterial strains and some of the extracts had a similar inhibition activity with the positive control. The fact that some of the juniper extracts showed a statistically significant difference with the Chloramphenicol inhibition activity may suggest promising evidence for further application of natural juniper extracts in different industrial fields. However, the initial concentration of the solutions must also be taken into consideration; the juniper samples’ dry extract in the antibacterial activity assay was 50 times higher than the antibacterial control. Based on the global characterization, *J. communis* berries and *J. oxycedurs* needle leaves showed higher antioxidant potential which, combined with their antibacterial activity, presents promising results for the utilization of these extracts for further applications in the food sector.

## Figures and Tables

**Table 1:** Moister content, water activity of the dried samples, and the extraction yield.

		Moisture Content	Water Activity	Extraction Yield	
		%	a_w_	EtA	Et
				%	%
Berries	*J. communis*	14.73 ± 0.00	0.52 ± 0.00	54.21	-
	*J. oxycedrus*	18.75 ± 0.00	0.64 ± 0.00	54.40	-
Leaves	*J. communis*	8.06 ± 0.00	0.26 ± 0.00	-	24.20
	*J. oxycedrus*	8.13 ± 0.00	0.24 ± 0.00	-	30.19

Average values for duplicate measurements ± standard deviation; EtA—ethanol 70% and acetic acid; Et—ethanol 70%.

**Table 2:** Global phytochemical characterization of juniper extracts.

	Berries		Leaves	
	*J. communis*	*J. oxycedrus*	*J. communis*	*J. oxycedrus*
TPC (mg GAE/g DW)	3.04 ± 0.09 ^A^	2.12 ± 0.05 ^B^	9.57 ± 0.99 ^a^	10.98 ± 0.58 ^a^
TFC (mg QE/g DW)	1.14 ± 0.36 ^A^	0.17 ± 0.09 ^B^	7.79 ± 0.08 ^b^	10.55 ± 0.24 ^a^
TAC (µg C3GE/g DW)	3.46 ± 0.00 ^B^	79.04 ± 0.00 ^A^	n.d.	n.d.
DPPH (mmol TE/g DW)	0.07 ± 0.01 ^A^	0.02 ± 0.00 ^B^	0.29 ± 0.03 ^a^	0.33 ± 0.00 ^a^
ABTS (mmol TE/g DW)	0.21 ± 0.01 ^A^	0.06 ± 0.00 ^B^	1.67 ± 0.01 ^b^	1.83 ± 0.01 ^a^

Average values for triplicates measurements ± standard deviation; n.d.—not detected. Uppercase letters, in the same row, are used for statistical comparisons between the berries; lowercase letters, in the same row, are used for statistical comparisons between the leaves. Means that do not share a letter are significantly different, based on the Tukey test (*p* > 0.05).

**Table 3:** HPLC characterization of bioactive compounds from juniper extracts.

Bioactive Compound (µg/g Extract)	Berries		Leaves	
	*J. communis*	*J. oxycedrus*	*J. communis*	*J. oxycedrus*
(−)—Epigallocatechin	n.d.	n.d.	n.d.	1129.23 ± 3.66 ^B^
Apigenin	2.67 ± 0.00 ^BC, B^	3.17 ± 0.01 ^K, A^	n.d.	n.d.
Caffeic acid	n.d.	n.d.	3.45 ± 0.19 ^O, b^	85.98 ± 0.28 ^D, a^
Caffeine	n.d.	n.d.	5.09 ± 0.05 ^M, b^	12.44 ± 0.08 ^L, a^
Catechin	93.98 ± 0.37 ^A^	n.d.	n.d.	n.d.
Chlorogenic acid	n.d.	10.74 ± 0.37 ^F^	n.d.	n.d.
Ellagic acid	0.02 ± 0.00 ^C, B^	445.69 ± 0.96 ^A, A^	8133.83 ± 4.03 ^A, a^	2890.05 ± 0.29 ^A, b^
Epicatechin gallate	8.00 ± 0.03 ^BC^	n.d.	n.d.	n.d.
Ferulic acid	n.d.	n.d.	23.66 ± 0.52 ^E, b^	87.84 ± 0.21 ^C, a^
Gallic acid	n.d.	290.23 ± 0.13 ^B^	38.28 ± 0.01 ^D^	n.d.
Hesperidin	n.d.	n.d.	6.04 ± 0.95 ^L^	n.d.
Kaempferol	10.83 ± 0.40 ^B, B^	63.30 ± 0.91 ^C, A^	179.87 ± 2.26 ^B, a^	57.76 ± 9.47 ^F, b^
Luteolin	n.d.	5.26 ± 0.08 ^H^	8.02 ± 0.40 ^H, a^	5.35 ± 0.66 ^O, b^
Myricetin	n.d.	28.24 ± 4.57 ^D^	n.d.	22.15 ± 0.19 ^I^
Naringin	n.d.	n.d.	12.13 ± 0.06 ^F, a^	8.26 ± 0.14 ^N, b^
*p*-Coumaric acid	n.d.	n.d.	n.d.	12.73 ± 0.09 ^K^
Procyanidin A1	n.d.	26.18 ± 0.06 ^E^	40.83 ± 0.09 ^C, b^	81.82 ± 0.05 ^E, a^
Protocatechuic acid	8.20 ± 1.16 ^BC^	n.d.	7.55 ± 0.09 ^J, b^	15.80 ± 0.75 ^J, a^
Quercetin 3-D-galactoside	6.07 ± 0.08 ^BC, A^	1.09 ± 0.03 ^L, B^	n.d.	56.45 ± 0.66 ^G^
Quercetin 3-glucoside	n.d.	5.05 ± 0.05 ^I^	7.17 ± 0.18 ^K, a^	2.52 ± 0.29 ^P, b^
Quercetin 3-β-D-glucoside	n.d.	n.d.	9.38 ± 0.11 ^G^	n.d.
Syringic acid	5.75 ± 0.02 ^BC^	n.d.	3.86 ± 0.01 ^N, b^	8.62 ± 0.02 ^M, a^
Theaflavin	2.74 ± 0.04 ^BC, B^	3.75 ± 0.02 ^J, A^	n.d.	n.d.
Vanillic acid	n.d.	9.88 ± 0.02 ^G^	7.66 ± 0.04 ^I, b^	23.21 ± 0.16 ^H, a^

Average values for duplicate measurements ± standard deviation; n.d.—not detected. Uppercase letters in black, in the same column, are used for statistical comparisons between the different compounds in one sample; letters in red, uppercase for berries and lowercase for leaves in the same row, are used for statistical comparisons between the samples. Means that do not share a letter are significantly different based on the Tukey test (*p* > 0.05) or the Games–Howell test (*p* < 0.05).

**Table 4:** Agar Well Diffusion Assay results from ethanolic extracts.

		Diameter Inhibition Zone (mm)/Tested Bacterial Strains		
		*Bacillus* spp.	*E. coli*	*S. aureus*
Berries	*J. communis*	25.75 ± 3.89 ^A, A^	17.50 ± 0.71 ^B, A^	26.00 ± 1.41 ^A, A^
	*J. oxycedrus*	20.25 ± 0.35 ^A, B^	19.00 ± 1.41 ^AB, B^	26.50 ± 2.12 ^A, A^
Leaves	*J. communis*	23.50 ± 2.12 ^a, a^	18.50 ± 0.71 ^b, a^	18.50 ± 0.71 ^b, a^
	*J. oxycedrus*	17.50 ± 0.71 ^a, b^	20.75 ± 0.35 ^ab, a^	19.50 ± 0.71 ^b, ab^
Controls	Chloramphenicol	22.00 ± 1.41 ^A, a^	22.00 ± 0.71 ^A, a^	30.00 ± 0.71 ^A, a^
	Solvent	n.d.	n.d.	n.d.

Average values for duplicate measurements ± standard deviation; n.d.—not detected; Well’s diameter—11 mm. Uppercase letters in black, in the same column, are used for statistical comparisons between the berries and the positive control; lowercase letters in black, in the same column, are used for statistical comparisons between the leaves and the positive control; letters in red, in the same row, are used for statistical comparisons between the different bacterial strains in one sample. Means that do not share a letter are significantly different based on the Tukey test (*p* > 0.05).

**Table 5:** Minimal inhibitory and bactericidal concentration of the ethanolic extracts.

		MIC (mg/mL)			MBC (mg/mL)		
		*Bacillus* spp.	*E. coli*	*S. aureus*	*Bacillus* spp.	*E. coli*	*S. aureus*
Berries	*J. communis*	0.05	6.25	6.25	12.50	12.50	12.50
	*J. oxycedrus*	0.05	6.25	6.25	12.50	12.50	12.50
Leaves	*J. communis*	n.d.	n.d.	25.00	n.d.	n.d.	n.d.
	*J. oxycedrus*	n.d.	n.d.	25.00	n.d.	n.d.	n.d.
Controls	Chloramphenicol	−	−	−	−	−	−
	Solvent	+	+	+	+	+	+

n.d.—not detected; (−)—no visible bacterial growth; (+)—visible bacterial growth.

## Data Availability

Data is contained within the article.
